# Assessing the impact of exact reads on reducing the error rate of read mapping

**DOI:** 10.1186/s12859-018-2432-7

**Published:** 2018-11-06

**Authors:** Farzaneh Salari, Fatemeh Zare-Mirakabad, Mehdi Sadeghi, Hassan Rokni-Zadeh

**Affiliations:** 1 Mathematics and Computer Science Department, Amirkabir University of Technology (Tehran polytechnic), Tehran, Iran; 2 School of Biological Science, Institute for Research in Fundamental Sciences (IPM) P.O. Box: 19395-5746, Tehran, Iran; 3 National Institute of Genetic Engineering and Biotechnology, Tehran, Iran; 4 Department of Biotechnology and Molecular Medicine, Zanjan University of Medical Sciences, Zanjan, Iran

**Keywords:** Reference-based assembly, Read mapping, Multi-mapping reads

## Abstract

**Background:**

Nowadays, according to valuable resources of high-quality genome sequences, reference-based assembly methods with high accuracy and efficiency are strongly required. Many different algorithms have been designed for mapping reads onto a genome sequence which try to enhance the accuracy of reconstructed genomes. In this problem, one of the challenges occurs when some reads are aligned to multiple locations due to repetitive regions in the genomes.

**Results:**

In this paper, our goal is to decrease the error rate of rebuilt genomes by resolving multi-mapping reads. To achieve this purpose, we reduce the search space for the reads which can be aligned against the genome with mismatches, insertions or deletions to decrease the probability of incorrect read mapping. We propose a pipeline divided to three steps: ExactMapping, InExactMapping, and MergingContigs, where exact and inexact reads are aligned in two separate phases. We test our pipeline on some simulated and real data sets by applying some read mappers. The results show that the two-step mapping of reads onto the contigs generated by a mapper such as Bowtie2, BWA and Yara is effective in improving the contigs in terms of error rate.

**Conclusions:**

Assessment results of our pipeline suggest that reducing the error rate of read mapping, not only can improve the genomes reconstructed by reference-based assembly in a reasonable running time, but can also have an impact on improving the genomes generated by *de novo* assembly. In fact, our pipeline produces genomes comparable to those of a multi-mapping reads resolution tool, namely MMR by decreasing the number of multi-mapping reads. Consequently, we introduce EIM as a post-processing step to genomes reconstructed by mappers.

## Background

The advent of next generation sequencing (NGS) technologies by greatly increasing the volume of produced data, created a genomic revolution. Massive amount of data and low cost of these technologies make it possible to determine large parts of a genome sequence in a short time. Today, biological research on any organism from viruses and bacteria to humans depends on the genome sequence information. In addition, sequences of organisms have an important role in understanding diseases.

In order to reconstruct a genome sequence based on NGS data, genome assembly, one of the challenging problems in bioinformatics, is defined. There are two different approaches to model genome assembly: *de novo* and reference-based assembly. In the first model, a novel genome sequence is reconstructed from scratch by only applying NGS reads. In the second one, a reference genome is employed to assemble the NGS reads by mapping them onto the reference.

Because of the large volume of NGS reads, established alignment algorithms such as Smith-Waterman aren’t efficient for read mapping. To reduce search space, several algorithms have been developed [1–5] using the seed-and-extending approach in which the reads are mapped onto the reference in two main steps. Firstly, some subsequences of each read are selected as seeds to find their positions in the reference. In this way, the candidate locations of the reads are determined rapidly. Secondly, each read is aligned to its candidate locations by a dynamic programming algorithm in order that the actual mapping positions are obtained.

During the past years, various algorithms have been designed to improve the accuracy and efficiency of mappers [6–13]. Although these algorithms represent appropriate approaches to reduce the time and space complexity, resolving multi-mapping reads in genome reconstruction has remained a challenge. Due to repetitive regions within the genome, some reads can be mapped to multiple locations of the reference genome. Multi-mapping reads may be aligned at incorrect locations since the read set contains sequencing errors and genetic variations relative to the reference. As a result, some errors such as mismatches and indels (insertions or deletions) are introduced to the reconstructed genome. Read mappers often randomly select one of the locations for a multi-mapping read as the primary one. Recently, a post-processing tool (MMR) has been developed [14] to find optimal locations for multi-mapping reads within DNA- and RNA-seq alignment results. It resolves the problem based on the assumption of aligned reads coverage uniformity.

In this study, we introduce a new view to resolving multi-mapping reads by increasing the rate of reads aligned uniquely to the reference in order to decrease the error rate of the reconstructed genome sequence. For this aim, we divide the reads into two groups in accordance with the reference genome. The idea is inspired by the following fact.

Consider a target genome (the genome from which a set of reads is sampled) which is highly similar to the respective reference genome. If the read set is mapped onto the reference, high percentage of the reference can be covered by the reads uniquely aligned without mismatches and indels (exact reads). Leftover alignable reads (inexact reads) are then mapped to the remaining parts of the reference. Therefore, to reconstruct most of the target genome, it is enough to find the locations of reads which have unique exact-matching with the reference. The rest of the target genome can be rebuilt by aligning remaining reads against the reference with mismatches and indels.

Most of the existing read mappers don’t consider any differences between the mapping of exact and inexact reads. For example, hash-based mappers find seeds which support mismatches (space-seeds) and gaps on the whole reference genome for all reads [15]. On the one hand, consecutive seeds are enough for exact reads and using space-seeds leads to excessive memory consumption. On the other hand, inexact reads are aligned by finding candidate locations on the whole reference genome, while according to high similarity between a target genome and its reference, searching in small parts of the reference is sufficient to find these types of reads.

Based on defining reads in two types: exact and inexact reads, we present a pipeline (EIM - mapping Exact and Inexact reads separately and then Merging the constructed contigs) for resequencing of a genome. To assess our pipeline, we have chosen Bowtie2 [7] as a highly cited and user-friendly mapper and used some real and simulated read sets. For a more complete evaluation of EIM pipeline, two other mappers are also used. Our results illustrate that EIM pipeline improves the quality of genomes reconstructed by the mappers in terms of error rate and yields comparable results to MMR in reducing errors.

## Methods

Let *S*=*s*_1_*s*_2_…*s*_*L*_ denote a DNA sequence in which ∀_1≤*i*≤*L*_ *s*_*i*_∈{*A*,*C*,*G*,*T*,*N*}; and |*S*| denote the length of *S*. A genome sequence is a long DNA sequence. A set of paired reads is defined as $R=\{\langle r_{1},r^{\prime }_{1}\rangle,\langle r_{2},r^{\prime }_{2}\rangle,\ldots,\langle r_{m},r^{\prime }_{m}\rangle \}$ where for each *i*, *r*_*i*_ and $r^{\prime }_{i}$ are short DNA sequences with length of k.

**Fig. 1:**
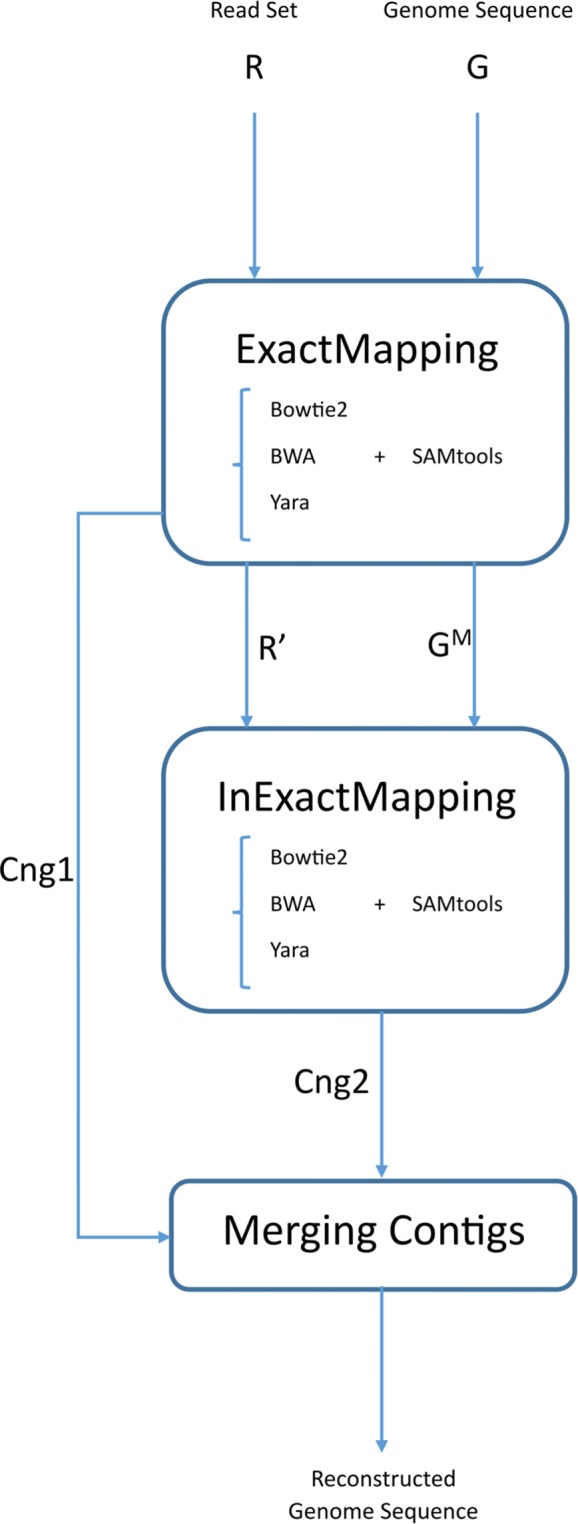
EIM pipeline overview and applied tools. The first step has three outputs: leftover reads (*R*^′^), modified remaining parts of the genome sequence (*G*^*M*^) and exact contigs (*Cng*1). The output of the second step is inexact contig set indicated by *Cng*2. Currently, EIM can apply one of mappers Bowtie2 [7], BWA [8, 9] and Yara [10] for mapping reads
  The set of reads is mapped onto the genome sequence without mismatches and indels. Then an exact contig set called *Cng*1 is generated from uniquely mapped reads.
ii. **InExactMapping**
  The remaining reads from previous step are mapped onto the regions of the genome which are covered with no contigs of *Cng*1 to construct an inexact contig set named *Cng*2.
iii. **MergingContigs**
  The two contig sets, *Cng*1 and *Cng*2 are merged to build up ultimate contigs.

We propose a three-step pipeline (Fig. 1) for reference-based assembly as below, where a set of paired reads *R* and a genome sequence *G* are given as inputs:

i. **ExactMapping**

In the following, each step of EIM pipeline is described in detail.

### ExactMapping

In this step, we should apply a mapper to align the set of reads with the genome without mismatches and indels. In this regard, the genome *G* and the read set *R* are given to the mapper as inputs. After running the mapper, two outputs are produced: *i*) set *R*^′^⊂*R* containing unmapped and multi-mapping reads *ii*) SAM file [16] including the information of the alignment. Then consensus sequence *C* is built up from uniquely mapped reads in the SAM file, where *C* is a DNA sequence with length |*G*|. Afterwards, a set of contigs called *Cng*1 is generated by breaking the sequence *C* at each position of ‘N’.

### InExactMapping

In this stage, genome sequence *G*=*g*_1_*g*_2_…*g*_*n*_ is modified based on consensus sequence *C*=*c*_1_*c*_2_…*c*_*n*_ to generate a new genome called *G*^*M*^. To construct genome *G*^*M*^, the following steps are taken:

1. Make sequence $ C^{\prime }= c^{\prime }_{1}c^{\prime }_{2} \ldots c^{\prime }_{n} $ as follows:
  $$c^{\prime}_{i} =\left\{ \begin{array}{ll} N & c_{i} \in \{ A,C,G,T\}, \\ g_{i} & c_{i}=N, \end{array}\right. $$
  where *C*^′^ contains all parts of genome *G* covered with no contigs of *Cng*1.
2. Generate sequence $ G^{M}= g^{M}_{1}g^{M}_{2}\ldots g^{M}_{n} $ by extending each contiguous nucleic acid sequence as:
  $$g^{M}_{i} =\left\{ \begin{array}{ll} c^{\prime}_{i} & c^{\prime}_{i} \in \{A,C,G,T\}, \\ g_{i} & c^{\prime}_{i}=N \& \exists_{j=1}^{k} c^{\prime}_{i \pm j} \in \{A,C,G,T\}, \\ N & o.w, \end{array}\right. $$
  where *k* is equal to the read length.

Then *G*^*M*^ is broken at each position of ‘N’, and as a result a set of contigs is obtained. After that, a mapper is used in order to align *R*^′^ against the set of contigs with mismatches and indels. Finally, a consensus sequence is made from mapped reads in the SAM file for each contig and added to *Cng*2.

### MergingContigs

In this part, the two contig sets *Cng*1 and *Cng*2 generated respectively at the steps of ExactMapping and InExactMapping, are combined to rebuild the target genome. Although *Cng*1 contains large contigs which make up most of the target genome, *Cng*2 is required to produce larger contigs including the differences with genome *G*. We merge the contig sets without alignment because the positions of contigs relative to the genome G are known. In this way, every two contigs of *Cng*1 are joined by a contig of *Cng*2 overlapping with both of them. Merging method is described in more detail below.

The union of *Cng*1 and *Cng*2 contig sets is defined as $Cng=\left \{\prec D^{i},s^{i},e^{i},t^{i} \succ \mid D^{i}=d^{i}_{1}d^{i}_{2}\cdots d^{i}_{e^{i}-s^{i}+1}\right \}$ where for each *i*, *D*^*i*^ is a contig belonging to either *Cng*1 or *Cng*2. The start and end positions of contig *D*^*i*^ on the reference are shown by *s*^*i*^ and *e*^*i*^, repectively. It should be noted that *s*^*i*^<*s*^*i*+1^ and *e*^*i*^<*e*^*i*+1^. Moreover, the value of *t*^*i*^ is set to 1 (or 2) when *D*^*i*^∈*Cng*1 (or *D*^*i*^∈*Cng*2). In the following, all the contigs in *Cng* are merged by a recursive equation: 
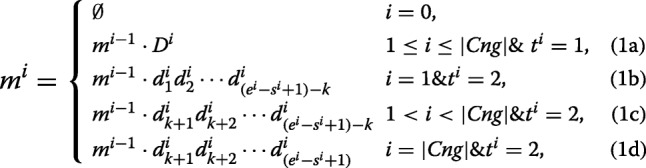
 where *k* is equal to the length of a read, and |*Cng*| is the number of contigs in the *Cng* set. For each i, *m*^*i*^ denotes the merged sequence achieved by combining *D*^1^ to *D*^*i*^. Part (1a) of the above equation shows that each contig of *Cng*1 has to be completely inserted to the merged sequence as it is highly probable that the contig has been made correctly. Parts (1b), (1c) and (1d) indicate how to insert a contig of *Cng*2 to the merged sequence after removing the extended parts (with length of *k*). The ultimate merged sequence is represented by *m*^|*Cng*|^ which may include some Ns because of *Cng*2 contigs. Thus *m*^|*Cng*|^ sequence is broken at each position of ‘N’ for generating output contigs of EIM pipeline.

### Datasets

Several real and simulated datasets are used to evaluate the accuracy of EIM pipeline. The first real dataset is an Illumina MiSeq pair-end read set from *E. coli* downloaded from [17, 18] which consists of about 1.5 million paired reads of 151 base-pair (bp) with coverage depth 100×. We apply *Escherichia coli str. K12 substr. MG1655* [GenBank:NC_000913] as a reference genome and *Escherichia coli O145:H28 str. RM12581* [GenBank: CP007136.1] as a related strain.

The second dataset includes four human chromosome read sets: Chr1, Chr10, Chr14 and Chr21 extracted from samples. The whole human genome samples are downloaded from the SRA database of National Center for Biotechnology Information (NCBI) with accession numbers SRR67780, SRR67785, SRR67787, SRR67789, SRR67791, SRR67792, SRR67793. The human reference genome GRCh38 is downloaded from [19]. All read sets contain 101 bp paired reads with the properties shown in Table 1.

**Table 1:** Real data sets properties

Data set	GenomeLength	Reads#	Coverage
Human Chr 1	248,956,422	80,623,200	34×
Human Chr 10	133,797,422	45,121,800	34×
Human Chr 14	107,349,540	36,117,398	42×
Human Chr 21	46,709,983	18,941,800	42×

We simulate several read sets for a prokaryotic and eukaryotic genome: *E. coli* and Arabidopsis thaliana. To simulate reads for *E. coli*, we create four genome sequences, E. coli-Mut1 to E. coli-Mut4 derived from *E. coliK12*. Then Illumina read sets, ReadSet1 to ReadSet8 and ReadSet9 to ReadSet12 are simulated for mutated genomes by DWGSIM [20] and ART [21], respectively. E. coli-Mut1 and E. coli-Mut2 have single nucleotide variants (SNVs) with the rate of 0.1%. E. coli-Mut2 has SNVs of random size among 1 to 3. E. coli-Mut3 has SNVs and deletions of the rates 0.09% and 0.01% respectively. E. coli-Mut4 has SNVs and insertions of the rates 0.09% and 0.01% respectively. The read sets, ReadSet1 to ReadSet4 are simulated such that the length and coverage depth of the reads are similar to those of the real read set from *E. coli K12* genome (i. e. more than 1.5 million paired reads of 150 bp). The read sets, ReadSet5 to ReadSet12 are simulated with low coverage (i. e. about 3000 paired reads of 150 bp) and sequencing error. The properties of simulated reads are shown in Table 2.

**Table 2:** Simulated data sets properties

Data set	Target genome	Genome length	Indel+SNV %	# SNVs	# Insertions	# Deletions	Read length	Coverage	Simulator
ReadSet1	E. coli-Mut1	4639675	0.1	4640	0	0	150	100×	DWGSIM
ReadSet2	E. coli-Mut2	4639675	0.1	4640	0	0	150	100×	DWGSIM
ReadSet3	E. coli-Mut3	4639138	0.1	4205	0	537	150	100×	DWGSIM
ReadSet4	E. coli-Mut4	4640180	0.1	4135	505	0	150	100×	DWGSIM
ReadSet5	E. coli-Mut1	4639675	0.1	4640	0	0	150	20×	DWGSIM
ReadSet6	E. coli-Mut2	4639675	0.1	4640	0	0	150	20×	DWGSIM
ReadSet7	E. coli-Mut3	4639138	0.1	4205	0	537	150	20×	DWGSIM
ReadSet8	E. coli-Mut4	4640180	0.1	4135	505	0	150	20×	DWGSIM
ReadSet9	E. coli-Mut1	4639675	0.1	4640	0	0	150	20×	ART
ReadSe10	E. coli-Mut2	4639675	0.1	4640	0	0	150	20×	ART
ReadSet11	E. coli-Mut3	4639138	0.1	4205	0	537	150	20×	ART
ReadSet12	E. coli-Mut4	4640180	0.1	4135	505	0	150	20×	ART

To generate reads for Arabidopsis thaliana, we create a genome sequence derived from TAIR10 [GenBank: CP002684.1-CP002688.1] reference genome. Firstly, TAIR10 genome sequence is mutated based on bur-0 strain variations obtaining from [22]. Then an Illumina read set including 15.6 million paired reads of 150 bp with coverage depth of 20× is simulated by ART simulator.

### Tools

Some tools are utilized for running EIM pipeline as follows. We use DWGSIM [20] and ART [21] for simulating reads, Bowtie2, Yara [10] and BWA [8, 9] for mapping reads, and SAMtools [16] for making consensus sequences. We also implement a simple hash-based aligner called ExactMapper for mapping reads without mismatches and gaps to make the pipeline faster.

The assessments on large genomes including human chromosomes and Arabidopsis thaliana are performed on a desktop which has a 3.60GHz Intel(R) Core(TM) *i*7−6850K 6-core processor and 32GB of RAM running 64-bit Ubuntu 18.04 LTS. The other assessments are performed on a laptop with an Intel(R) Core(TM) *i*7−3517U processor and 8GB of RAM running 64-bit Ubuntu 15.10.

At ExactMapping step, we apply ExactMapper aligner for small genomes to generate a SAM file and extract remaining reads (unmapped and multi-mapping reads) simultaneously. Next, SAM file is given to a script to build up a consensus sequence C from uniquely mapped reads. At InExactMapping step, we employ one of the aforementioned mappers with appropriate parameters and then construct the consensus sequence by SAMtools. For this purpose, The ‘*--keep-masked-ref*’ parameter is set for ‘*bcftools call*’ command of SAMtools to be able to make consensus in IUPAC positions of the reference genome.

It should be noted, for large genomes such as the human chromosome 14, we use Bowtie2 in ExactMapping step. The ‘*--score-min*’ parameter of Bowtie2 is set to the value ‘ *C*,0,−1’ to only map the reads with exact matches to the genome. Th unmapped and multi-mapping reads are extracted from the SAM file by a script and the consensus sequence is constructed by SAMtools.

### Evaluation metrics

To evaluate EIM pipeline, we calculate some *contiguity* and *quality* metrics by QUAST [23] for contig sets (genomes) reconstructed by ExactMapping step, EIM and the mappers.

We use two metrics to compare the contiguity of the contig sets as follows:

- **Contigs-500**: The number of contigs with length of greater than 500 bp belonging to the contig set.
- **N50**: The length of the smallest contig in the set that contains the fewest (largest) contigs whose combined length represents at least 50% of assembly [24].

We use quality metrics for indicating the *accuracy* of the reconstructed genomes. To calculate some quality metrics, each set of contigs is aligned to the target (or reference) genome to find the number of errors regarding to each contig set as below:

- **Errors**: The total number of mismatches and indels (insertions and deletions) in the aligned contigs relative to the target genome.
- **IUPAC-codes**: The total number of IUPAC ambiguity positions in the contig set.
- **Genome-Fraction**: The percentage of the target (or reference) genome covered by the aligned contigs.

when the target genome is not available, we apply the following quality measure to test the accuracy of the reconstructed genomes.

- **Remapped-Reads**: The percentage of the reads which are identically mapped (i.e. without mismatches and indels) onto the contigs.

## Results

A set of reads and a reference genome are given to EIM pipeline as inputs and then EIM constructs a set of contigs as output by stepwise mapping of the reads onto the reference. The sequencing errors and genetic differences as well as repetitive regions in the genome are the factors which introduce mapping errors such as mismatches and indels into the contigs relative to the target genome.

To evaluate the results of EIM pipeline, we use different datasets in terms of similarity between the target and reference genomes as follows:

1. By considering a reference genome identical to the target genome, we initially assess our pipeline where the real read set from *E. coli* *K*12 includes sequencing errors.
2. According to the high similarity between any human genome and the human reference, we investigate results of EIM pipeline where the real reads from a human chromosome 14 contain sequencing errors as well as SNVs. It is to be noted that the target genome is not available.
3. By simulating some target genomes highly similar to *E. coli* *K*12 genome, we examine EIM pipeline in which the simulated reads include SNVs. In this way, we can test the accuracy of EIM more precisely since the target genomes are available.
4. By using a closely related genome to *E. coli* *K*12 as a reference, we perform EIM pipeline on a real read set from *E. coli* *K*12 to assess our pipeline where the similarity between the target and reference genomes is not very high.

For completing the evaluation of EIM, we apply different mappers on a real read set from *E. coli* *K*12 and a closely related genome to it as a reference, and then compare the results of EIM pipeline to the respective mappers. In addition, we evaluate our pipeline on eukaryotic genomes of human and Arabidopsis thaliana.

### Assessment of EIM on a real dataset of *E. coli K12*

To test the accuracy of EIM, we examine the effect of sequencing errors without considering any other factors. For this purpose, *E. coli K12* genome and its reads generated by using Illumina are given to EIM as inputs. Accordingly, the target and reference genomes are the same and the read set includes sequencing errors.

An Illumina sequencer has an error rate of <0.1*%* [25], because of which only 61.79% of the reads can be mapped at the first step of EIM pipeline (ExactMapping). However, contigs constructed from the uniquely mapped reads cover nearly entire of the target genome (99.995% in Table 3). At the second step of our pipeline (InExactMapping), remaining reads from the first step are mapped onto just 0.005% of the reference. As shown in Table 3, the last step of EIM (MergingContigs) produces a contiguous contig including 2 errors, while Bowtie2 mapper makes 11 contigs containing the same number of errors on this sample data. Although Bowtie2 generates more contigs than EIM, the Genome-Fraction values of both contig sets are the same (100%) because the gaps between contigs of Bowtie2 are too small compared to the total length of the target genome.

**Table 3:** Real datasets analysis where the inputs of EIM pipeline are the read set and reference genome

Assembly	Exact	EIM	Bowtie2
*E. coli*			
Contigs-500	64	1	11
N50 (kbp)	1250.9	4639.673	597.8
Errors	0	2	2
Genome-Fraction (%)	99.995	100	100
Human chromosome 14			
Contigs-500	27312	451	1179
N50 (kbp)	6	407.6	174.1
Errors	18	44407	44911
Genome-Fraction (%)	93.22	99.80	99.71

The evaluation metrics has been defined in the text. The columns headed ’Exact’, ’EIM’ and ’Bowtie2’ represent the contiguity and quality of contigs constructed by ExactMapping step of EIM, EIM and Bowtie2, respectively

This assessment shows that contig sets reconstructed by EIM and Bowite2 are the same in terms of accuracy when the read set contains sequencing errors.

### Assessment of EIM on a real dataset of human chromosome 14

In this assessment, our goal is to investigate the accuracy of EIM where the set of reads extracted from a genome includes sequencing errors as well as SNVs and indels relative to the reference. We perform EIM on the human chromosome 14 reference and the reads from a human chromosome 14.

Due to sequencing errors and genetic differences between human genomes, only about half of reads (58.33%) are aligned at the ExactMapping step. The contigs constructed from this volume of the reads cover 93.22% of the chromosome 14 reference (Table 3). Furthermore, the results presented in Table 3 show that EIM makes significantly fewer contigs than Bowtie2. In other words, the comparison of N50 values indicates that EIM can make a contig set more contiguous than that of Bowtie2. Moreover, the contigs of EIM include fewer errors relative to the reference than those of Bowtie2. Although comparing with the reference genome gives insight into the error rate of the reconstructed genomes, some differences are true differences rather than errors. Since the target genome is not available, we use the read set to assess the accuracy of EIM. In this way, the reads are mapped without mismatches and indels to the reconstructed genomes to calculate Remapped-Reads values. The results of the remapping show that the Remapped-Reads values for the genomes reconstructed by EIM and Bowtie2 are 60.87% and 58.68% respectively. This is an appropriate evidence that the reconstructed genome by EIM is more accurate than that of Bowtie2.

Our results show that when the target and reference genomes are highly similar, EIM pipeline can reconstruct a more accurate genome than the one rebuilt by Bowtie2 mapper.

### Assessment of EIM on simulated data

To assess the accuracy of EIM more precisely, the target genome sequences are required. Since target sequences are typically not available for most of individuals and strains, we use simulated data. To do so, we make some genome sequences derived from *E. coli K12* genome by creating mismatches and indels using different rates and then simulate read sets from the mutated genomes (Table 2).

We test EIM pipeline on ReadSet1 (Table 2) and *E. coli* *K*12 as a reference genome. To compare contigs generated by EIM and Bowtie2, we align both contig sets against E. coli-Mut1 (the target genome) and present the results in the second, third and last columns of Table 4. Although EIM pipeline rebuilds a contiguous contig, it introduces more errors than Bowtie2. It is also worth mentioning that the contigs of ExactMapping step of EIM called *Exact* contigs have 90.285% Genome-Fraction value which in comparison with that obtained by real data experiment (99.995% in Table 3) is very low. It seems that a lower Genome-Fraction value of Exact contigs leads to the higher errors in the final contigs produced by EIM.

**Table 4:** Simulated ReadSet1 analysis where the inputs of EIM pipeline are the read set and either the reference genome (ref) or the genome reconstructed by Bowtie2 (cns-bt)

c 1	c 2	c 3	c 4	c 5	c 6	c 7
Assembly	Exact	EIM	Exact	EIM (*v*1)	EIM (*v*2)	Bowtie2
	/ref	/ref	/cns-bt	/cns-bt	/cns-bt	/ ref
Contigs-500	2817	1	68	9	2	6
N50 (kbp)	1.82	4639.6	156.4	1108.9	3543.3	2385.650
Errors	3	45	0	28	29	38
Genome-Fraction (%)	90.285	100	99.381	99.992	99.999	100

The evaluation metrics has been defined in the text. The columns headed ’Exact/ref’, ’EIM/ref’, ’EIM (*v*1)/cns-bt’, ’EIM (*v*2)/cns-bt’ and ’Bowtie2’ represent the contiguity and quality of the respective contigs. Also *E. coli* K12 genome is denoted by ’ref’ and the consensus sequence constructed by Bowtie2 on *E. coli* genome is denoted by ’cns-bt’

We need to point out that the more fraction of the target genome is covered by Exact contigs, the smaller parts of the reference remain for InExactMapping step of EIM. Hence the probability that the leftover reads are aligned at true locations is increased and as a result, the error rate of the reconstructed genome is reduced. Furthermore, the fraction of the target genome covered by Exact contigs is directly proportional to the similarity between the target and reference genomes. In other words, the higher similarity between the target and reference genomes leads to fewer errors in the genome reconstructed by EIM pipeline. Accordingly, since the genome sequence reconstructed by a mapper is more similar to the target genome than to the reference (*ref*), the genome sequence reconstructed by Bowtie2 (*cns-bt*) is fed to EIM instead of the reference as input.

The results can be seen in the fourth and fifth columns of Table 4. The comparison of the second and fourth columns shows that by giving the genome sequence reconstructed by Bowtie2 instead of the reference sequence to EIM as the input, the Genome-Fraction value of Exact contigs increases from 90 to >99%. In addition, the number of errors in final contigs of EIM decreases from 45 to 28. It suggests that the genome sequence reconstructed by a mapper is a better input for our pipeline as it leads to a lower error rate. Our analysis up to this point shows that by feeding *cns-bt* instead of *ref* to EIM pipeline as input, the error rate is reduced. It is important to note that the error rate decreasing is valuable only when EIM rather maintains the same *N*50 and Genome-Fraction values as those of the input genome. However, the results of EIM in the fifth column compared to the last column of Table 4 indicate that this condition is not satisfied.

We observed that *cns-bt* includes 137 IUPAC-codes while *ref* contains no IUPAC-codes. Furthermore, the genome reconstructed by mapping a read set onto a reference sequence containing IUPAC-codes is less contiguous than the reference because SAMtools makes a consensus sequence including ‘N’ in the IUPAC-code positions. Thus the existence of IUPAC-codes in the input genome of EIM yields a more fragmented genome as output. To solve this issue, we execute SAMtools with a parameter allowing to build consensus in the IUPAC-code positions instead of substituting ‘N’ ambiguity character (“Tools” subsection). As shown in the sixth column of Table 4, EIM with this modification makes contigs which in addition to including less errors than *cns-bt* (the input genome), are nearly as contiguous as *cns-bt* and with high coverage of the target genome. In the following, EIM described in the fifth and sixth columns of Table 4 are considered as versions one (*v*1) and two (*v*2), respectively.

Tables 5 and 6 represent the results of applying EIM (*v*2) pipeline and Bowtie2 mapper to the simulated read sets with high and low coverage, respectively. As illustrated by the results, not only can EIM (*v*2) decrease the error and IUPAC-code rates, but it can also maintain the contiguity and Genome-Fraction value very close to Bowtie2.

**Table 5:** Simulated high coverage datasets analysis where the inputs of EIM pipeline are the read set and genome reconstructed by Bowtie2

Assembly	Exact	EIM (*v*2)	Bowtie2
ReadSet1			
Contigs-500	68	2	6
N50 (kbp)	156.4	3543.3	2385.6
Errors	0	29	38
IUPAC-codes	0	11	137
Genome-Fraction (%)	99.381	99.999	100
ReadSet2			
Contigs-500	142	3	5
N50 (kbp)	62.7	1371.6	939.1
Errors	0	45	92
IUPAC-codes	0	29	246
Genome-Fraction (%)	99.338	100	99.997
ReadSet3			
Contigs-500	96	6	3
N50 (kbp)	94.4	1096.2	3267.7
Errors	2	54	87
IUPAC-codes	0	11	140
Genome-Fraction (%)	99.285	99.997	100
ReadSet4			
Contigs-500	77	3	3
N50 (kbp)	115.5	2337.4	1530.2
Errors	6	55	72
IUPAC-codes	0	15	34
Genome-Fraction (%)	99.436	99.998	100

The evaluation metrics has been defined in the text. The columns headed ’Exact’, ’EIM (*v*2)’ and ’Bowtie2’ represent the contiguity and quality of contigs constructed by ExactMapping step of EIM, EIM (*v*2) and Bowtie2, respectively

**Table 6:** Simulated low coverage datasets analysis where the inputs of EIM pipeline are the read set and genome reconstructed by Bowtie2

Assembly	Exact	EIM (*v*2)	Bowtie2		Exact	EIM (*v*2)	Bowtie2
DWGSIM simulator				ART simulator			
ReadSet5				ReadSet9			
Contigs-500	172	14	58		137	13	56
N50 (kbp)	43.9	735.7	159.6		75.2	909.5	175.3
Errors	0	53	64		1	45	53
IUPAC-codes	0	36	137		0	38	102
Genome-Fraction (%)	98.899	99.991	99.983		98.912	99.983	99.965
ReadSet6				ReadSet10			
Contigs-500	163	6	64		178	18	63
N50 (kbp)	44.4	1698.5	112.2		45.4	485.2	116.9
Errors	1	68	92		2	68	83
IUPAC-codes	0	28	95		0	39	187
Genome-Fraction (%)	98.843	99.998	99.976		98.871	99.988	99.984
ReadSet7				ReadSet11			
Contigs-500	424	11	55		425	17	70
N50 (kbp)	16.6	590.9	125.9		18	386.6	115.4
Errors	2	185	361		6	179	369
IUPAC-codes	0	24	117		0	38	151
Genome-Fraction (%)	98.666	99.993	99.985		98.697	99.989	99.983
ReadSet8				ReadSet12			
Contigs-500	397	17	56		366	13	49
N50 (kbp)	18.9	493.4	141.1		21.6	529.5	127.6
Errors	8	190	331		8	186	322
IUPAC-codes	0	21	105		0	23	121
Genome-Fraction (%)	98.771	99.99	99.979		98.799	99.983	99.982

The evaluation metrics has been defined in the text. The columns headed ’Exact’, ’EIM (*v*2)’ and ’Bowtie2’ represent the contiguity and quality of contigs constructed by ExactMapping step of EIM, EIM (*v*2) and Bowtie2, respectively. The results of running the pipeline on datasets simulated by DWGSIM and ART are shown in left and right side of the table, respectively

The results of this assessment show that our pipeline can improve the genome sequence reconstructed by Bowtie2 mapper in terms of accuracy when a highly similar reference to the target genome is available and the read set includes SNVs relative to the reference.

### Assessment of EIM on a real dataset of *E. coli K12* and a closely related genome

In this assessment, we examine the accuracy of EIM when similarity between the target and reference genomes is not so high. The application is where a reference is not available and a closely related genome is used as a reference. We apply *E. coliO145:H28* as a closely related genome to *E. coliK12*.

To evaluate EIM on the read set from *E. coliK12*, a genome sequence is reconstructed from mapping the reads onto *E. coliO145:H28* genome by Bowtie2, then the reconstructed genome and the reads are given to EIM as inputs. Table 7 shows that the contig sets generated by EIM (*v*1) and EIM (*v*2) contain fewer errors and IUPAC-codes than that of Bowtie2. Moreover, EIM (*v*2) can make contigs which have nearly the same Genome-Fraction value and N50 size as those of Bowtie2.

**Table 7:** Real dataset analysis where a closely related genome is used as a reference

Assembly	Exact	EIM (*v*1)	EIM (*v*2)	Bowtie2	MaSuRCA	EIM (*v*1) + MaSuRCA
Contigs-500	497	334	263	259	114	114
N50 (kbp)	12.3	22.2	32	29.3	106	106
Errors	58	618	1190	2472	2407	1786
IUPAC-codes	0	17	56	280	0	5
Genome-Fraction (%)	87.013	87.811	88.578	88.575	99.058	99.058

The evaluation metrics has been defined in the text. The columns headed ’Exact’, ’EIM (*v*1)’, ’EIM (*v*2)’, ’Bowtie2’, ’MaSuRCA’ and ’EIM (*v*1) + MaSuRCA’ represent the contiguity and quality of contigs constructed by ExactMapping step of EIM, EIM (*v*1), EIM (*v*2), Bowtie2, MaSuRCA and combining the contig sets of EIM (*v*1) and MaSuRCA assembler, respectively

It should be noticed that the Genome-Fraction values of the contigs produced by EIM and Bowtie2 are less than 90*%*. In such cases where there is no reference available and the related genome is not highly similar to the target genome, *de novo* genome assembly is a better approach for reconstructing the genome sequence. However, the genome sequences generated by *de novo* assemblers are not error-free. For this reason, approaches for improving the accuracy of *de novo* assembled contigs are needed. Here we use the contigs generated by EIM to improve the contigs produced by a *de novo* assembler. In fact, we use version one of EIM pipeline because contigs of EIM (*v*1) include less errors than those of EIM (*v*2). The read set is assembled by MaSuRCA [26], one of the best assemblers at GAGE-B [27], then the contigs constructed by EIM and MaSuRCA are combined into a contig set including fewer errors than the contigs of MaSuRCA (Table 7).

This analysis indicates that when a closely related genome is used as a reference, and thus the reference and target genomes are not highly similar, EIM (*v*2) can reconstruct a genome sequence with the same contiguity and Genome-Fraction value including less errors and IUPAC-codes than the genome reconstructed by Bowtie2 mapper. In addition, the genome rebuilt by EIM (*v*1) can decrease the error rate of a genome sequence generated by a *de novo* assembler such as MaSuRCA.

### Evaluation of EIM by different mappers

To evaluate the performance of our pipeline by using mappers other than Bowtie2, we select BWA as a popular and widely used mapper and Yara as one of the state-of-the-art mappers. We use the three mappers and version 2 of EIM on the read set from *E. coliK12* and *E. coliO145:H28* genome as a reference. For each mapper, the genome reconstructed by the mapper is given to EIM (*v*2) as input and the mapper itself is applied for aligning reads in the second step of EIM (*v*2) (i. e. InExactMapping).

**Fig. 2:**
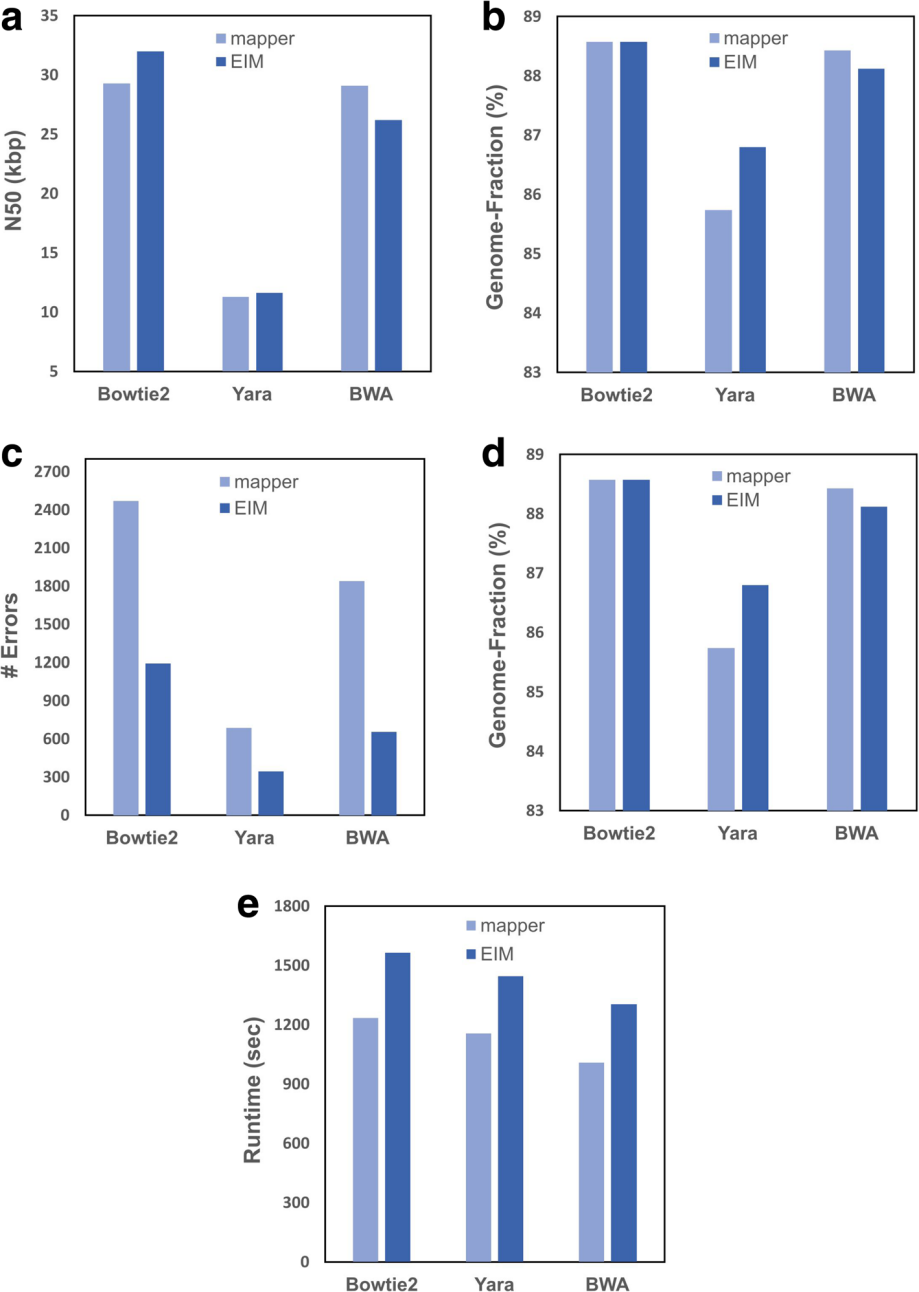
The comparison of contigs generated by Bowtie2, Yara and BWA with the respective contigs of EIM on the real read set of E. coli *K12*. Firstly, the mappers were executed on the read set and the reference, and then the contig sets were generated. Secondly, for each mapper, EIM (*v*2) was run on the read set and the contig set constructed by the mapper while using it at the second step for mapping. Finally, the contiguity and quality of contigs were computed as **a** N50 size **b** Genome-Fraction value **c** The number of errors **d** The number of IUPAC codes. In addition, the running time of obtaining contigs was measured and showed in seconds (**e**)

As illustrated in Fig. 2, for all mappers, EIM pipeline maintains N50 size and Genome-Fraction value close but not identical to those of the mappers (Fig. 2a and b). It also reduces the number of errors and significantly decreases the number of IUPAC-codes (Fig. 2c and d).

Figure 2e shows the running times of the three mappers compared to EIM. Since the input genome of EIM is built by a mapper, the running time of reconstructing a genome by EIM is the total of mapper and EIM pipeline runtimes. In addition, the running time of reconstructing a genome by a mapper is the total of read mapping and consensus constructing runtimes, which the second one is more time-consuming. Our pipeline decreases the computational time of making a consensus by a two-step mapping. In ExactMapping, most of the reads are exactly aligned and a SAM file is made from which the consensus sequence can be constructed by a simple and fast script without using SAMtools. Moreover, only a low percentage of reads is transferred to InExactMapping step and thus the consensus sequence is made rapidly by SAMtools in this stage. Consequently, the overhead time of reconstructing the *E. coli* genome by EIM pipeline after running a mapper is less than one-third of that of the respective mapper (Fig. 2e).

This evaluation demonstrates that EIM pipeline can be used as a post-processing tool to improve the genome reconstructed by a mapper to a more accurate one in an acceptable runtime while maintaining the contiguity and Genome-Fraction value of the input genome.

### Evaluation of EIM on de novo assembled genomes

In this section, we assess the effect of EIM pipeline on the results of de novo assemblies. For this purpose, we compare EIM with Pilon framework [28] and Columbus module of Velvet assembler [29]. These tools get a draft or reference genome and mapped reads on it, to apply read mappings for improving genome assembly.

In the following, we first generate two genomes by Velvet and MaSuRCA assemblers on the real read set from *E. coliK12*. Then each draft genome is inputted to EIM, Pilon, and Columbus.

**Fig. 3:**
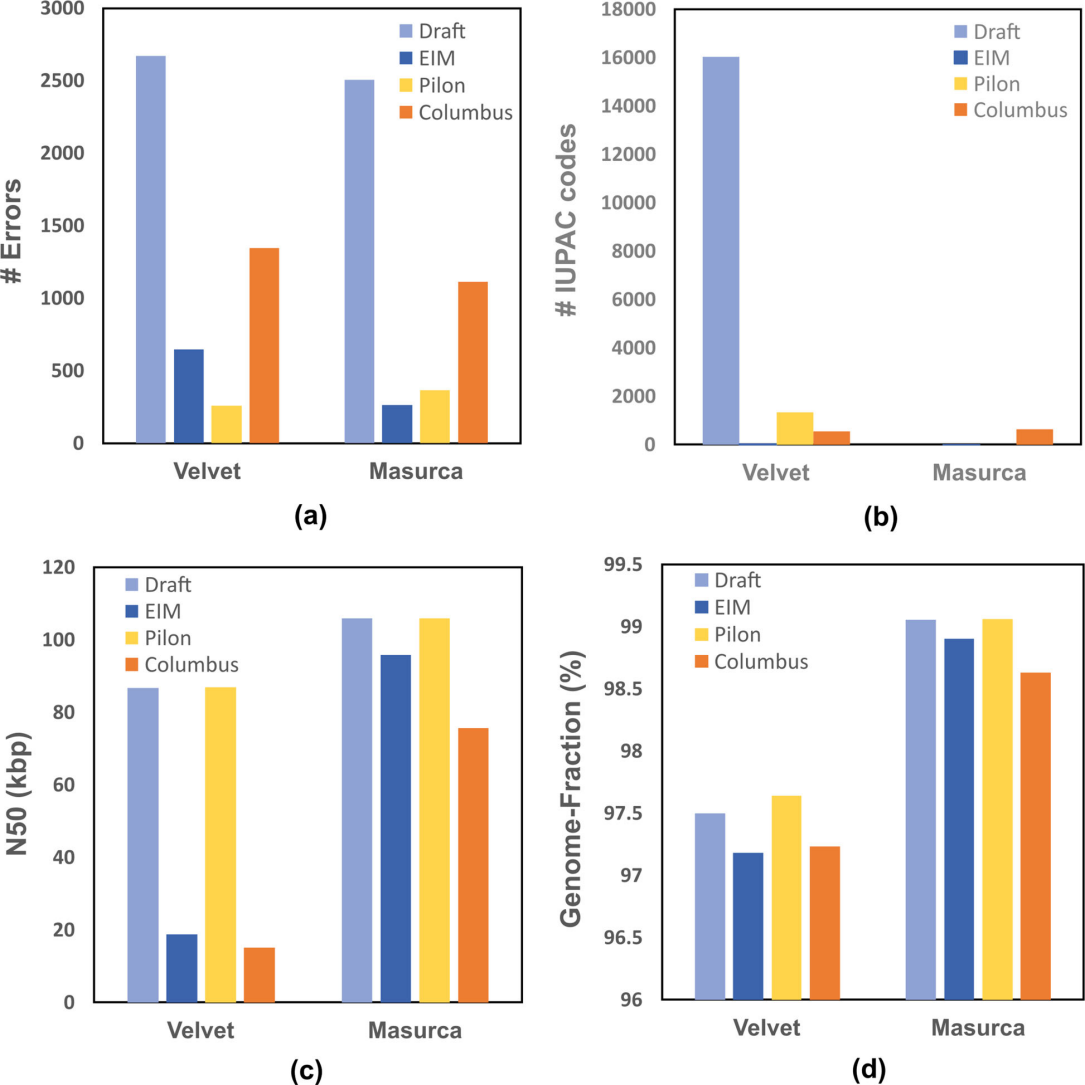
The comparison of contigs generated by EIM, Pilon and Columbus on the real read set of E. coli *K12*.Firstly, two draft genomes were generated by Velvet and MaSuRCA *de novo* assemblers. Secondly, EIM, Pilon and Columbus were run by each draft and mapped reads on the draft. Finally, the quality and contiguity of contigs were computed as **a** The number of errors **b** The number of IUPAC codes **c** N50 size **d** Genome-Fraction value

As illustrated in Fig. 3, all frameworks reduce the number of errors and dramatically decrease the number of IUPAC-codes when that of the draft genome is too high (Fig. 3a and b). Although EIM and Columbus decrease N50 size (Fig. 3c), they maintain Genome-Fraction value close to those of draft genomes (Fig. 3d).

The results of this comparison show that EIM pipeline has an impact on reducing the error rate of the genomes generated by *de novo* assembly.

### Evaluation of EIM on eukaryotic genomes

For the final evaluation, we run EIM pipeline on the datasets of human as a mammalian and Arabidopsis thaliana as a model plant. To evaluate EIM on human, we select the smallest and the largest chromosomes as well as a chromosome with average length namely Chr21, Chr1, and Chr10, respectively and extract the reads of each one from real samples of the whole human genome. Then we run EIM on each dataset separately. For evaluating our pipeline on Arabidopsis thaliana, we simulate a dataset for all chromosomes of bur-0 strain and use TAIR10 as the reference to run EIM.

As shown in Table 8, EIM pipeline reduces error rates on all three human chromosomes and bur-0 strain of Arabidopsis. To be precisely measured the accuracy of generated contigs, the reads are exactly mapped onto each contig set to calculate Remapped-Reads value. As seen, EIM increases Remapped-Reads values. Furthermore, the results show that our pipeline considerably increases the N50 size of contig sets generated for human chromosomes because of the high similarity between human genomes.

**Table 8:** Evaluating EIM on some eukaryotic datasets

Assembly	EIM (*v*2)	Bowtie2
Human chromosome 1		
Contigs-500	2497	5018
N50 (kbp)	420.7	158.2
Errors	115381	120726
IUPAC-codes	7862	158247
Genome-Fraction (%)	99.828	99.614
Remapped-Reads (%)	52.02	50.95
Human chromosome 10		
Contigs-500	1443	2478
N50 (kbp)	399.9	149.2
Errors	70478	73842
IUPAC-codes	5508	112333
Genome-Fraction (%)	99.209	99.034
Remapped-Reads (%)	51.72	49.93
Human chromosome 21		
Contigs-500	1239	2362
N50 (kbp)	237.8	101
Errors	22904	23579
IUPAC-codes	3232	46155
Genome-Fraction (%)	99.114	97.73
Remapped-Reads (%)	44.58	42.23
Arabidopsis Thaliana (bur-0)		
Contigs-500	6936	6987
N50 (kbp)	428.8	417.4
Errors	136539	179312
IUPAC-codes	4842	2370
Genome-Fraction (%)	98.634	98.572
Remapped-Reads (%)	66.32	65.24

The evaluation metrics has been defined in the text. The columns headed ’EIM (*v*2)’ and ’Bowtie2’ represent the contiguity and quality of contigs obtained based on the results of EIM (*v*2) and Bowtie2, respectively

In order to examine the effect of different chromosomal regions on accuracy of EIM, we test our pipeline on portions of a human chromosome. To achieve this goal, we divide Chr1, the largest human chromosome, to twenty five same-length regions as follows:

$$P = \{p_{1}, \ldots, p_{25}\} \;\; for \;\; each \;\; i \;\; |p_{i}| \simeq 10 Mbp. $$

**Fig. 4:**
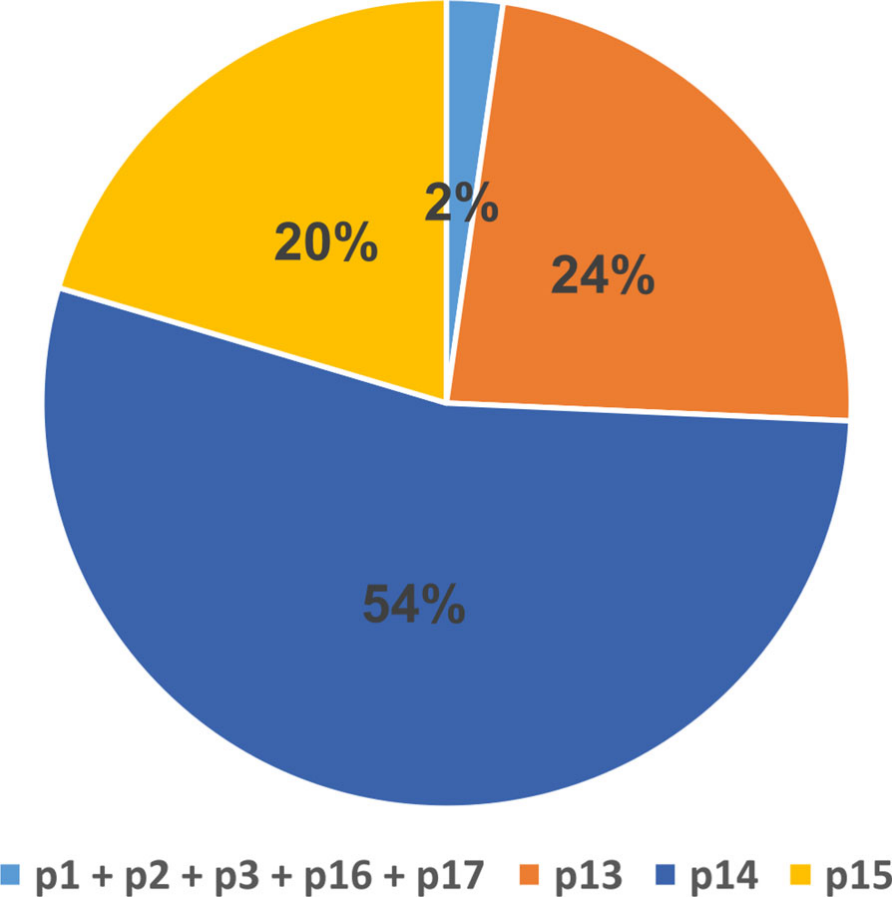
The distribution of N characters in the regions of Chr1. The regions of *p*_14_, *p*_13_ and *p*_15_ have 54*%*, 24*%* and 20*%* of N characters of Chr1, respectively. The centromere consists of *p*_13_, *p*_14_ and *p*_15_ regions which contain 97*%* Ns of Chr1

The number of ambiguity characters (Ns) is assessed in each *p*_*i*_1≤*i*≤25 (Fig. 4). We omit *p*_14_ because this region is a whole sequence of Ns. We then run EIM on the read set of Chr1 and each *p*_*i*_1≤*i*≤25*andi*≠14, separately.

**Fig. 5:**
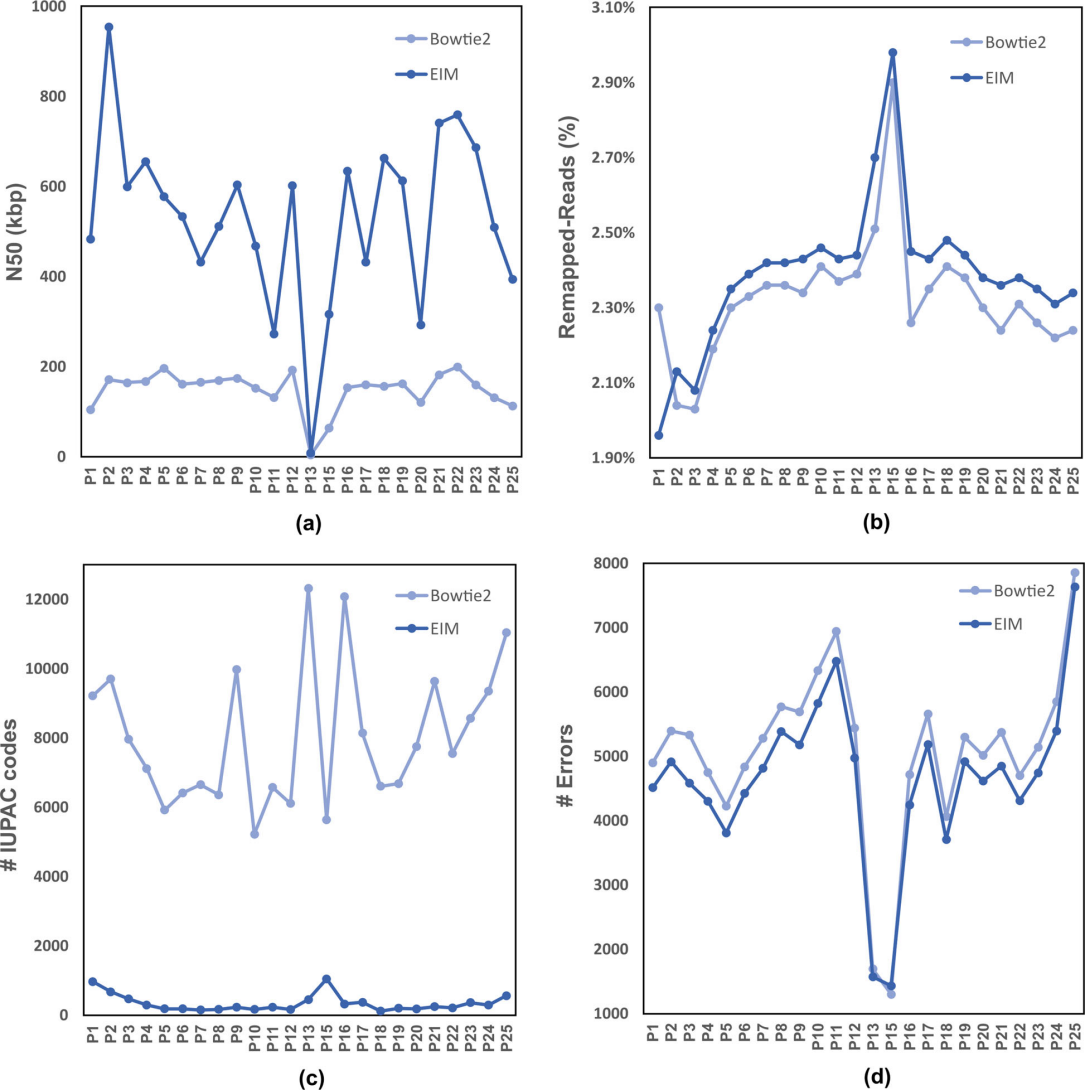
The comparison of contigs generated by Bowtie2 and EIM on the regions of Chr1. Firstly, Chr1 was divided to some regions, *p*_*i*_1≤*i*≤25. Secondly, Bowtie2 and EIM were run by the read set of Chr1 and each *p*_*i*_1≤*i*≤25*andi*≠14, separately. Finally, the quality and contiguity of contigs were computed as **a** N50 size **b** The Remapped-Reads value **c** The number of IUPAC codes **d** The number of errors

As shown in Fig. 5, EIM pipeline increases N50 values and reduces error numbers, and significantly decreases IUPAC numbers for all regions. Note that, because of the high fraction of Ns in centromere region, contigs generated by Bowtie2 and EIM on *p*_13_ and *p*_15_ have low N50 size and low error numbers (Fig. 5a and d).

**Fig. 6:**
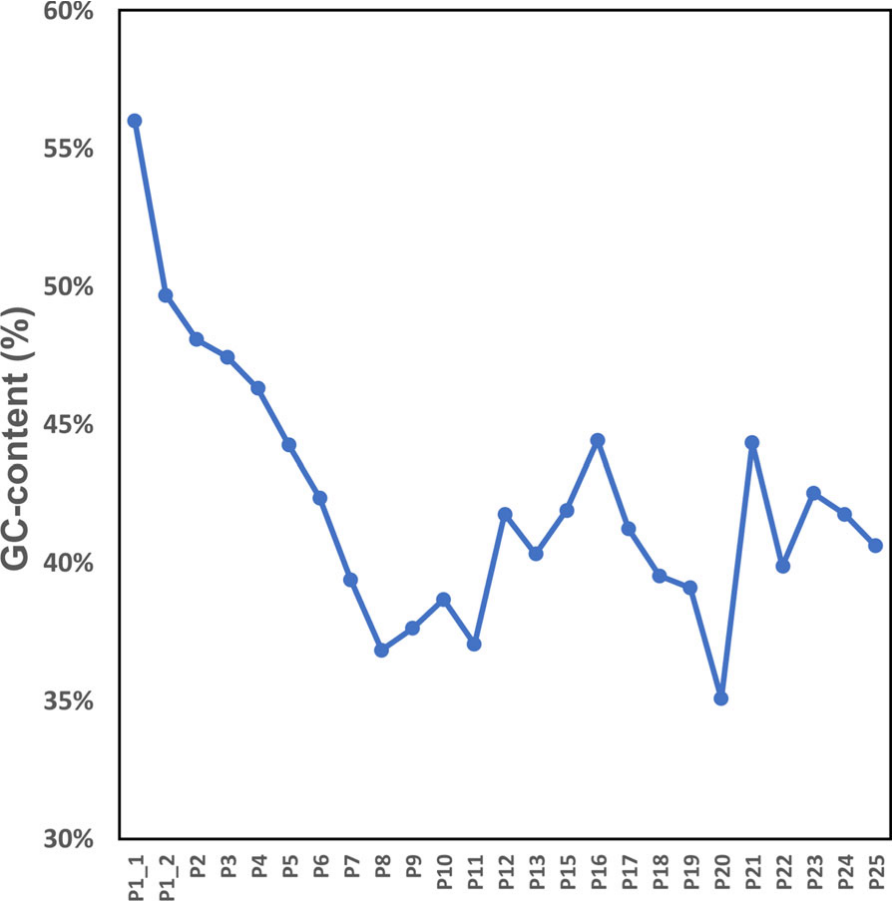
GC-content of contig sets generated by EIM on the regions of Chr1. As shown, $p_{1\_1}$ has the maximum GC-content among all regions

In addition, EIM increases Remapped-Reads values for all regions except for the first one (Fig. 5b). To explore the reason, we break the *p*_1_ region from Ns and select two of five yielded portions called $p_{1\_1}$ (∼2.1 Mbp) and $p_{1\_2}$ (∼7.2 Mbp) for analysis because their length is more than 1 Mbp. Then we run EIM on the read set of Chr1 and $p_{1\_1}$ and $p_{1\_2}$ regions, separately. The results show that the Remapped-Reads value of contigs generated by EIM is 0.05*%* more than that of Bowtie2 for $p_{1\_2}$ while this value is 0.35*%* less than that of Bowtie2 for $p_{1\_1}$. Thus the shorter portion i.e. $p_{1\_1}$ leads to decreasing of the Remapped-Reads value of *p*_1_ region. According to this observation, we examine GC-content of all regions of Chr1. The GC-content of $p_{1\_1}$ is 56*%* while GC-content of $p_{1\_2}$ and other regions are less than 50*%* (Fig. 6).

The results from GC-content analysis suggest that running EIM on genomic regions with less than 50*%* GC-content can generate contigs which are more accurate than those of a mapper.

## Discussion

As mentioned in the “Background” section, one of the most challenging aspects of genome sequence reconstruction from NGS data is the existence of multi-mapping reads. We claim that EIM pipeline decreases the number of multi-mapping reads and thus reduces the error rate of the reconstructed genome. To demonstrate this claim, we analyse each step of EIM separately. Let the input genome sequence of EIM be the genome reconstructed by a mapper like Bowtie2.

At ExactMapping step, the consensus sequence is built from the reads uniquely mapped and thus the resulting Exact contigs contain very low errors (see the ‘Exact’ column in Tables 6 and 7). Therefore the number of errors in the contigs of the next step plays a determining role in the error rate of the genome reconstructed by EIM. The reads not applied in this step, namely multi-mapping and unmapped reads are transferred to the second step to be aligned with mismatches and indels.

**Fig. 7:**
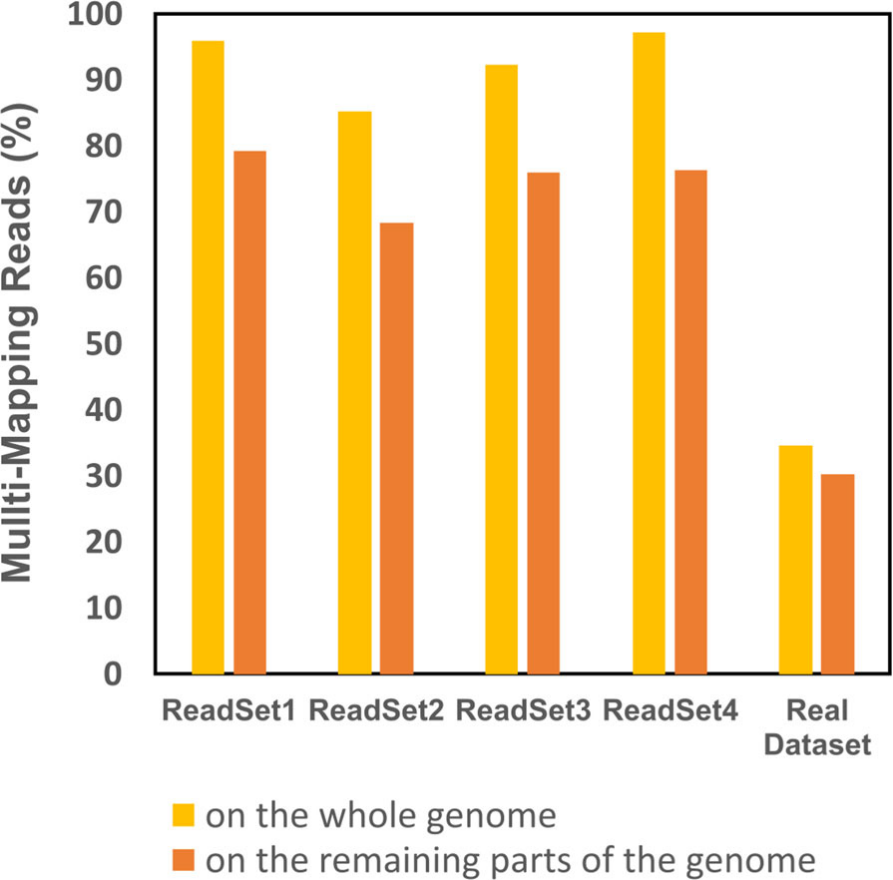
Multi-mapping reads on the whole and the remaining parts of the genome. A real and four simulated datasets were used. The orange and yellow bars show the percentage of multi-mapping reads where the reads were aligned against the whole genome, and in which the reads were mapped onto the regions not covered by the contigs of the first step of EIM, respectively

At InExactMapping step, the remaining reads are aligned to the parts of the input genome not covered by any Exact contigs and then the consensus sequence is generated. To examine the effect of EIM on multi-mapping reads, we should compare the number of multi-mapping reads in this step to that obtained by mapping the reads onto the whole input genome. To do so, the reads that can be mapped at the second step of EIM, are aligned again to the whole input genome. Figure 7 shows that our pipeline leads to less multi-mapping reads on the simulated and real datasets. In fact, on the simulated datasets, EIM can decrease the number of multi-mapping reads by finding unique mapping locations for 17*%* of them on average.

To complete the examination of the effect of EIM pipeline on multi-mapping reads, EIM is compared to a multi-mapping reads resolution tool, MMR. We compare the genome reconstructed by EIM to the genome obtained based on the results of MMR on the read set from *E. coliK12* and *E. coliO145:H28* as a reference. In this way, firstly, the reads are mapped by Bowtie2 onto the reference and a SAM file is generated. Then a sorted BAM file and a consensus sequence are built from SAM file as the inputs of MMR and EIM, respectively. MMR produces a BAM file that assigns an optimal mapping location to each multi-mapping read, while EIM generates a contig set such that the number of multi-mapping reads are decreased.

As shown in Table 9, both approaches maintain the contiguity and reduce the error rate of the input. In addition, EIM can impressively decrease the number of IUPAC-codes from 280 to 56. The running time of reconstructing *E. coli* genome by EIM (330 sec) is significantly less than that of MMR (999 sec) without considering the running time of making the inputs. Note that for reconstructing a genome based on MMR results, a consensus construction stage is required after applying MMR which causes to increase the runtime.

**Table 9:** Comparing EIM and MMR results on a real dataset

Assembly	Bowtie2	EIM (*v*2)	MMR
Contigs-500	259	263	260
N50 (kbp)	29.3	32	31.3
Errors	2472	1190	1369
IUPAC-codes	280	56	224
Genome-Fraction (%)	88.575	88.578	88.671

The evaluation metrics has been defined in the text. The columns headed ’Bowtie2’, ’EIM (*v*2)’ and ’MMR’ represent the contiguity and quality of contigs obtained based on the results of Bowtie2, EIM (*v*2) pipeline and MMR tool, respectively

As shown by this analysis, the results of EIM pipeline are comparable to a multi-mapping reads resolution tool in terms of the main goal, that is, reducing the error rate of the genome reconstructed by a mapper.

## Conclusion

The goal of our work is to improve the accuracy of contigs generated using NGS read mappers by decreasing their error rate. To achieve this purpose, we design EIM pipeline which aligns the exact and inexact reads against the genome sequence at two separate steps to map the inexact reads more precisely. The assessment of our pipeline on simulated and real read sets show that the separation of reads is effective in reducing the number of mismatch and indel errors with regard to the target genome and significantly decreases the number of IUPAC-codes in the input genome. The evaluation of EIM by three mappers namely Bowtie2, BWA and Yara also indicates that our pipeline, as a post-processing step to different mappers, can improve the genome sequences reconstructed by them in an acceptable running time. In addition, EIM pipeline can reconstruct a comparable genome to that of MMR (a multi-mapping reads resolution tool) in terms of error rate.

## Abbreviations

bp: Base-pair
BAM: Binary Alignment/Map
Chr: chromosome
cns-bt: Genome sequence reconstructed by Bowtie2
EIM: mapping Exact and Inexact reads separately and then Merging the constructed contigs
indel: Insertion or deletion
kbp: Kilo base-pair
NCBI: National Center for Biotechnology Information
NGS: Next generation sequencing
ref: Reference
SAM: Sequence Alignment/Map
SNV: Single nucleotide variant
SRA: Short read archive
